# Prevalence and cost of hospitalized patients with asymptomatic COVID-19 in 2020 in Spain

**DOI:** 10.3389/fpubh.2023.1229561

**Published:** 2023-08-01

**Authors:** Blanca Álvarez-del Río, Laura Sánchez-de Prada, Alejandro Álvaro-Meca, Marta Martín-Fernández, F. Javier Álvarez, Eduardo Tamayo, Eduardo Gutiérrez-Abejón

**Affiliations:** ^1^Departamento de Farmacología, Facultad de Medicina, Universidad de Valladolid, Valladolid, Spain; ^2^BioCritic, Grupo de Investigación Biomédica en Cuidados Críticos, Valladolid, Spain; ^3^Departamento de Microbiología e Inmunología, Hospital Clínico Universitario de Valladolid, Valladolid, Spain; ^4^Centro Nacional de Gripe de Valladolid, Valladolid, Spain; ^5^Centro de Investigación Biomédica en Red de Enfermedades Infecciosas (CIBERINFEC), Instituto de Salud Carlos III, Madrid, Spain; ^6^Departamento de Medicina Preventiva y Salud Pública, Universidad Rey Juan Carlos, Madrid, Spain; ^7^Dirección Técnica de Farmacia, Gerencia Regional de Salud de Castilla y León, Valladolid, Spain; ^8^Facultad de Empresa y Comunicación, Universidad Internacional de la Rioja (UNIR), Logroño, Spain

**Keywords:** asymptomatic, COVID-19, prevalence, costs, hospitalized, Spain

## Abstract

**Introduction:**

COVID-19 transmission has been characterized by the presence of asymptomatic patients. Additionally, most studies evaluating costs focus on symptomatic COVID-19 cases.

**Objective:**

To describe the prevalence, characteristics, and costs of asymptomatic COVID-19 cases at admission in Spanish hospitals in 2020.

**Methods:**

A nationwide study was performed, and data of hospitalized patients were collected of the Minimum Basic Data Set in Spain during 2020. Patients with COVID-19 codes as a primary and as a secondary diagnosis at admission were selected. Variables collected included age, sex, length of stay, in-hospital death, admission, length of stay and death in intensive care unit, mechanical ventilation and ventilatory assistance. COVID-19 related hospital costs were calculated using diagnosis-related groups from the Minimum Basic Data Set. Patients and costs were disaggregated by sex, age group, intensive care unit admission and epidemic wave (first or second) and main diagnosis.

**Results:**

A total of 14,742 patients were admitted with asymptomatic COVID-19 in Spanish hospitals representing 6.35% of all COVID-19 admitted patients. The total cost of admissions with asymptomatic COVID-19 was €105,933,677.6 with a mean cost per patient of €7,185.8 with higher mean cost in the first wave despite only 2.7% of cases were found during that time. Based on primary diagnosis, the higher number of cases of asymptomatic COVID-19 were found in “Pregnancy, childbirth and the puerperium” followed by “diseases of the circulatory system”.

**Conclusions:**

There was a high prevalence of asymptomatic cases during screening at admission process in Spanish hospitals in 2020. The highest number of cases was found among the group of “pregnancy, childbirth, and puerperium” followed by “diseases of the circulatory system.” The higher costs might be due not only to the main pathology at admission but to the associated healthcare provisions needed in case of positive COVID-19 testing.

## 1. Introduction

The COVID-19 (coronavirus disease 2019) pandemic caused by the severe acute respiratory syndrome-2 (SARS-CoV-2) coronavirus (1) has resulted in an overpowering macroeconomic impact, causing a decrease of 7.4% in gross domestic product in Europe (2). For healthcare systems, the initial economic hit was mainly supported by hospitals (3), with an overloaded use of Intensive Care Units (ICU) and in-hospital resources, such as mechanical ventilation, as the most expensive items (4).

In Spain, healthcare is free for public use, and is lawfully guaranteed by the National Health System, covering for 47.45 million people in 2020, although private healthcare providers are also available. The Spanish hospital network, accounted for 467 public and 310 private hospitals with a total 50 capacity of 153,265 beds, in 2020. During the emergency state, private hospitals were made available for public use. Moreover, at the peak of the first wave of the pandemic, more than 13,000 beds were opened for ICU admissions, three times the pre-pandemic provision (5). Actually, it has been estimated that the economic health care burden of COVID-19 hospitalized patients in Spain was €1.23 billions in 2020 (6).

Most COVID-19 studies focus on direct costs of COVID-19 symptomatic patients (4). However, the proportion of asymptomatic patients has been estimated to be high (7, 8). Those patients play an important role in the transmission within the community and healthcare facilities as they have been identified as a potential source of infection due to having similar viral loads as symptomatic patients (9, 10).

In this study, we aim to describe the prevalence, characteristics, and the cost of asymptomatic COVID-19 cases in hospitalized patients screened as part of an inpatient admission in 2020.

## 2. Methods

### 2.1. Study design

A nationwide population-based retrospective economic evaluation of hospitalizations with asymptomatic COVID-19 was conducted in Spanish hospitals during 2020. The study followed the CHEERS reporting guideline and was approved by the Ethics Review Board (CEIm Area de Salud Valladolid Este, PI 22-2855).

### 2.2. Measures

**Figure 1:**
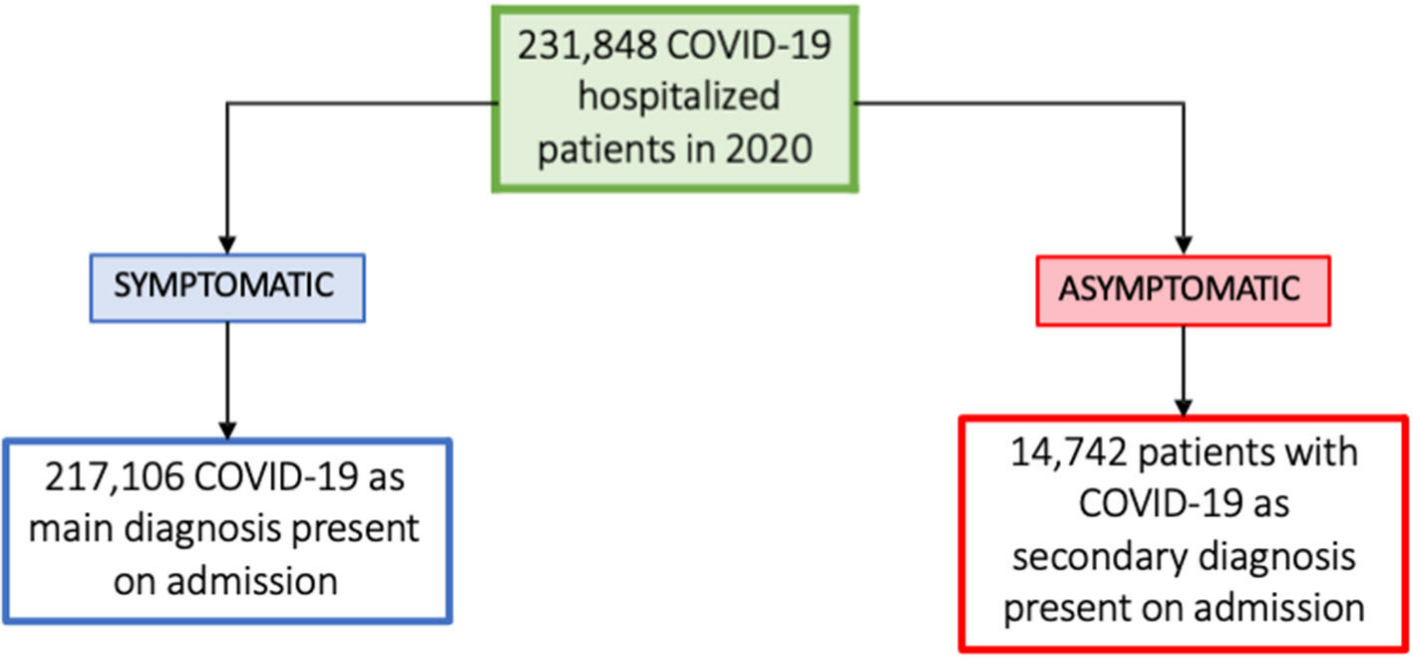
Flow chart of patient selection.

Data were collected from the Minimum Basic Data Set (MBDS) (11), obtained by the National Surveillance System for Hospital Data and published with 2 years lag by the Ministry of Health. The MBDS is a clinical and administrative database filled in at discharge covering for 99.5% of Spanish hospitals. Patients with COVID-19 codes B97.29 and U07.1 as the primary diagnosis at admission, on one hand, and as the secondary diagnosis at admission, on the other, following the International Classification of Diseases 10^th^ Revision, Clinical Modification (ICD-10-CM), were selected (12). Asymptomatic COVID-19 patients include those infected with the virus who do not experience any symptoms of the disease and would not realize they are infected, thus contributing to viral spread (13). Therefore, we considered COVID-19 asymptomatic cases when the hospitalized patients were admitted with a primary diagnosis other than COVID-19, but during inpatient admission screening COVID-19 were found to be positive (Figure 1).

Variables collected included age, sex, length of stay (LoS), in-hospital death, admission in ICU, ICU length of stay, ICU death, mechanical ventilation and ventilatory assistance. During 2020, SARS-CoV2 circulated in two waves in Spain, the first one since its until June 30^th^, 2020, and the second from July 1^st^, 2020, until December 31^st^, 2020 (5).

COVID-19 related hospital direct costs were calculated using diagnosis-related groups (DRG) from the MBDS (11). The GDR weights and costs are calculated from a representative sample of hospitals that perform analytic accounting in our country and are periodically collected in the Spanish Record of Hospital Costs. Patients and costs were disaggregated by sex, age group, intensive care unit (ICU) admission and epidemic wave (first or second), provinces, and main diagnosis following the International Classification of Diseases 10^th^ Revision, Clinical Modification (ICD-10-CM) were selected (12). All costs were expressed in euros (€) in 2020 values. The annual currency equivalence in 2020 was 1€ =1.1422$ United States dollars (USD) (14).

### 2.3. Statistical analysis

Results were reported as mean (95%CI) for continuous variables and as frequency (percentages) for categorical variables. Differences between groups were assessed using an unpaired, 2-tailed *t*-test; Mann-Whitney test; and Kruskal-Wallis test with Bonferroni correction adjustment for multiple comparisons (α = 0.05) for continuous variables when appropriate. Statistical analysis was conducted with Python 3.9 (Python). Two-sided *P* < 0.05 indicated statistical significance.

## 3. Results

A total of 14,742 patients were admitted with asymptomatic COVID-19 in Spanish hospitals (Table 1) and 217,106 patients admitted with a primary diagnosis of COVID-19 (6). Asymptomatic cases represent 6.35% of all COVID-19 hospitalized patients in 2020. From the 14,742 patients, 8,307 were females [56.3%] and 6,435 were males [43.7%], and the mean age of 59.0 [CI95%: 58.6–59.3] years old (Table 1). In-hospital mortality during 20202 was 10.6% and was similar in both waves (13.0% vs. 10.5%, *p* = 0.12). However, length of stay was 10.7 days but differed between waves with 57.8 days in the first compared to 9.4 days in the second (*p* < 0.001). Only 398 (2.7%) asymptomatic cases were found during the first wave.

**Table 1:** Hospitalization of asymptomatic COVID-19 cases and costs in 2020 in Spain.

**Description**	**No patients**	**Total cost (€)^a^**	**Mean cost(€) /patient (CI95%)^a^**	***P*-value**
	14,742	105,933,677.6	7,185.84 (7,080.98–7,290.71)	
**Epidemic wave**				
First	398	5,284,120.45	13,276.68 (11,789.13–14,764.24)	< 0.001
Second	14,344	100,649,557.15	7,016.84 (6,918.64–7,115.04)	
**Type of admission**				
General	13,569	87,266,111.79	6,431.29 (6,356.69–6,505.88)	< 0.001
ICU	1,173	18,667,565.81	15,914.38 (15,063.97–16,764.79)	
**Sex**				
Male	6,435	52,624,813.39	8,177.90 (7,994.27–8,361.54)	< 0.001
Female	8,307	53,308,864.21	6,417.34 (6,299.95–6,534.73)	
**Age group**				
< 40	4,176	23,079,625.79	5,526.73 (5,378.72–5,674.74)	< 0.001
40–59	2,515	20,059,792.48	7,976.06 (7,686.29–8,265.83)	
60–79	4,072	35,103,496.2	8,620.7 (8,362.17–8,879.23)	
>79	3,979	27,690,763.13	6,959.23 (6,824.6–7,093.86)	

^a^To convert to US dollars multiply by 1.14.

ICU, intensive care unit.

**Figure 2:**
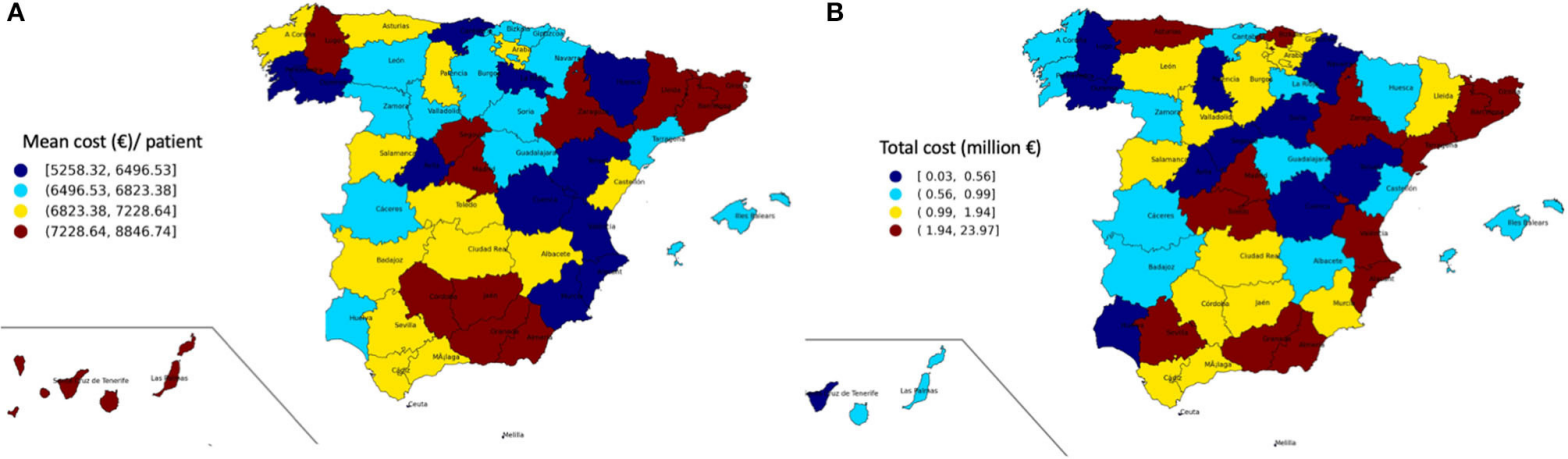
**(A)** Mean cost (€) per patient in each Spanish province. **(B)** Total cost per patient (€) in each Spanish province.

The total cost of admissions with asymptomatic COVID-19 was €105,933,677.6 with a mean cost per patient of €7,185.8, higher in the first wave (€13,276.7) compared to the second (€7,016.8, *p* < 0.001). Of these, 8.0% of patients required ICU and ICU-mortality reached 1.7%. Higher mean cost per patient was found in ICU patients with €15,914.4 compared to regular admission with €6,431.3 (p < 0.001). Costs were higher in males and in 60–79-year-old group (*p* < 0.001) (Table 1). Additionally, mean cost per patient and total cost by provinces in Spanish territory are described in Figure 2.

**Figure 3:**
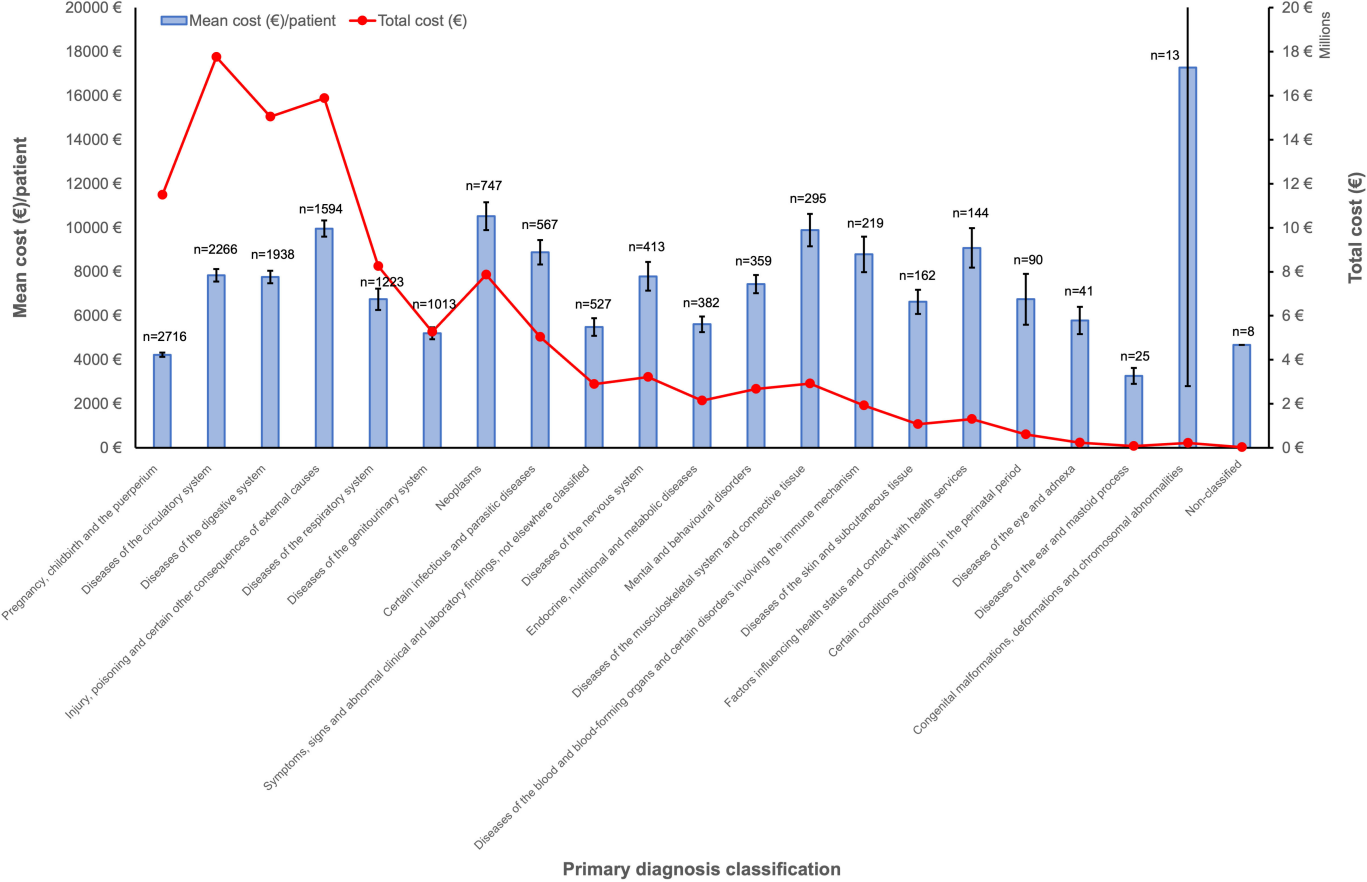
Costs of hospitalized patients with asymptomatic COVID-19 based on their primary diagnosis (ICD-10-CM) in 2020 in Spain. Columns represent mean cost per patient (€) with confidence of interval 95% (left axis). Dots represent total cost (right axis). The total number of patients per primary diagnosis group is indicated above each column.

Based on primary diagnosis, the higher number of cases of asymptomatic COVID-19 were found in “Pregnancy, childbirth and the puerperium” followed by “diseases of the circulatory system” (2,716 [18.4%] and 2,266 [15.4%] patients) (Figure 3). Despite the great variability observed in costs according to the primary diagnosis, the highest total cost was found in “diseases of the circulatory system” with a total of €17,756,914.27 (Table 2).

**Table 2:** Costs of hospitalized patients with asymptomtic COVID-19 based on their primary diagnosis (ICD-10-CM) in 2020 in Spain.

**No patients**	**Mean cost (€)/patient (CI95%)^a^**	**Total cost (€)^a^**	**Primary diagnosis classification**
2,716	4,231.63 (4,132.02–4,331.24)	11,493,106.35	Pregnancy, childbirth and the puerperium
2,266	7,836.24 (7,551.1–8,121.37)	17,756,914.27	Diseases of the circulatory system
1,938	7,758.58 (7,473.62–8,043.54)	15,036,131.78	Diseases of the digestive system
1,594	9,960.55 (9,596.14–10,324.96)	15,877,117.29	Injury, poisoning and certain other consequences of external causes
1,223	6,750.95 (6,269.92–7,231.99)	8,256,415.54	Diseases of the respiratory system
1,013	5,204.89 (4,938.47–5,471.31)	5,272,552.69	Diseases of the genitourinary system
747	10,524.9 (9,896.41–11,153.40)	7,862,103.05	Neoplasms
567	8,884.74 (8,331.00–9,438.49)	5,037,648.59	Certain infectious and parasitic diseases
527	5,487.29 (5,090.12–5,884.46)	2,891,801.62	Symptoms, signs and abnormal clinical and laboratory findings, not elsewhere classified
413	7,792.96 (7,141.11–8,444.81)	3,218,491.47	Diseases of the nervous system
382	5,615.96 (5,262.3–5,969.62)	2,145,296.83	Endocrine, nutritional and metabolic diseases
359	7,443.63 (7,029.27–7,857.98)	2,672,261.70	Mental and behavioral disorders
295	9,892.26 (9,153.32–10,631.20)	2,918,216.70	Diseases of the musculoskeletal system and connective tissue
219	8,788.43 (7,984.08–9,592.78)	1,924,666.33	Diseases of the blood and blood-forming organs and certain disorders involving the immune mechanism
162	6,633.06 (6,086.28–7,179.84)	1,074,556.14	Diseases of the skin and subcutaneous tissue
144	9,082.45 (8,184.81–9,980.09)	1,307,872.74	Factors influencing health status and contact with health services
90	6,749.51 (5,591.26–7,907.76)	607,455.85	Certain conditions originating in the perinatal period
41	5,787.6 (5,170.26–6,404.93)	237,291.40	Diseases of the eye and adnexa
25	3,268.17 (2,903.11–3,633.24)	81,704.36	Diseases of the ear and mastoid process
13	17,278.45 (2,807.62–31,749.27)	224,619.79	Congenital malformations, deformations and chromosomal abnormalities
8	4,681.64 (4,681.64–4,681.64)	37,453.09	Non-classified

^a^To convert to US dollars multiply by 1.14.

## 4. Discussion

In our national study, we found a 6.35% of asymptomatic COVID-19 patients among all COVID-19 hospitalized patients in Spain. A recent model has estimated the prevalence of asymptomatic patients in Madrid to be of 28% in the first three waves (15), however it accounted for the whole of population. Additionally, previous studies have estimated the prevalence to be 0.25% with 0.75% among in-hospital patients (8), focusing on hospitalized population but lower compared to our findings. It is noteworthy that most cases were found in “pregnancy, childbirth and the puerperium” and “diseases of the circulatory system.” Actually, estimates indicate that the percentage of asymptomatic cases among pregnant women could reach 54% (8) which could be a picture of how the relative immunosuppressive state of pregnancy could lead to more asymptomatic cases (16). Regarding the circulatory system, COVID-19 coagulopathy (17) and increased risk of myocarditis, pericarditis and cardiac arrythmias have been described upon COVID-19 infections (18).

Previous studies have assessed the direct costs of symptomatic COVID-19 in different contexts (6, 19, 20). However, economic information on asymptomatic COVID-19 is scarce. In our study, the evaluation of costs showed that patients admitted with asymptomatic COVID-19 had higher mean costs per patient (+26.4%) than those found in a previous study of hospitalized patients with symptomatic COVID-19 (6). Despite the small number of patients admitted in the first wave, the mean cost per patient during the first wave (€13,276.68) almost doubled mean compared to the second (€7,016.84). This could be the result of different circumstances. First, the observed cost increase could be attributable not only to the infection itself which could increase the severity of underlying conditions requiring increased expenses. Also, during the first wave which took place during lockdown only patients with highly severe conditions would dare to go to the hospital, while in the second, “normal” activity was reinstated. Additionally, during that period the skyrocketing price of medical supplies due to an impaired availability and high demand could have affected costs (21). A matched cohort study in the United States showed that COVID-19 increased the costs of all pathologies studied in hospitalized patients (19). Even though, we can't describe the exact increment for each primary diagnosis category, it seems that the high costs are a reflect of that. Further studies should clarify the specific cost contribution of asymptomatic COVID-19.

Our study has limitations as we performed a retrospective study using the Spanish MBDS. However, COVID-19 codes have shown high sensitivity and specificity in other countries (22). One limitation is that the patients included in the study had a high variability of primary diagnoses, and we were unable to assess and compare costs in similar patients without asymptomatic COVID-19. Another limitation is that the data available does not allow to compare costs between public and private hospitals. By contrast, the strengths of our study include being a nationwide study of all hospitalizations with asymptomatic COVID-19 infection. The compulsory screening before admission in Spain during 2020, has allowed us to provide a general vision of the frequency of asymptomatic cases at hospital admission and the relevant costs related to them in 2020 in the Spanish population, unlike studies in individual regions or hospitals.

To conclude the prevalence of asymptomatic COVID-19 cases in hospitalized patients screened at admission was high in 2020 in Spain. The highest number of cases was found in “pregnancy, childbirth, and puerperium” group, followed by “diseases of the circulatory system.” The higher cost of asymptomatic COVID-19 is probably related to the main pathology itself but also to the healthcare measures taken in case of asymptomatic COVID-19 positive patients. Although, further specific studies need to be performed in order to estimate the increased cost associated to asymptomatic infection.

## Data availability statement

The datasets presented in this article are not readily available because the MDBS is the property of the Ministry of Health. Therefore, any researcher can request the data related to this article from the Ministry of Health by email (icmbd@msssi.es), by fax (+34915964111), or by mail (Instituto de Información Sanitaria, Área de Información y Estadísticas Asistenciales, Ministerio de Sanidad, Consumo y Bienestar Social. Paseo del Prado 18–20; 28071 Madrid. Spain). Requests to access the datasets should be directed to Ministry of Health, icmbd@msssi.es.

## Ethics statement

The studies involving human participants were reviewed and approved by Ethics Committee of Valladolid East Health Area. Written informed consent from the participants' legal guardian/next of kin was not required to participate in this study in accordance with the national legislation and the institutional requirements.

## Author contributions

AÁ-M and ET had full access to all of the data in the study and take responsibility for the integrity of the data and the accuracy of the data analysis. BÁ-dR, MM-F, and EG-A conceived and design the study. FÁ and ET obtained the funding and supervised the work. LS-dP, AÁ-M, FÁ, ET, and EG-A performed data analysis and statistical analysis was performed by AÁ-M and LS-dP. BÁ-dR and LS-dP wrote the draft of the manuscript. The final version was revised and approved by all authors.
